# P03.01. Characteristics of residents and training sites influence successful completion of the Integrative Medicine in Residency program

**DOI:** 10.1186/1472-6882-12-S1-P254

**Published:** 2012-06-12

**Authors:** P Lebensohn, S Dodds, B Kligler, A Brooks, P Cook, V Maizes

**Affiliations:** 1 University of Arizona, Tucson, USA; 2 Beth Israel Medical Center, New York City, USA

## Purpose

To describe resident and training site characteristics influencing resident completion of a 200-hour curriculum in integrative medicine (IM).

## Methods

Resident and residency site characteristics were examined to determine factors influencing completion of the Integrative Medicine in Residency (IMR) curriculum for the 2011 graduating class. Completion criteria included finishing ≥80% of the online courses plus a final score on the medical knowledge test of ≥70%. Resident characteristics used as predictors included demographics; medical school type [US MD, DO, foreign medical graduates (FMG)]; and, responses to a post-match survey administered before the residency began in 2008 on previous participation in CAM courses or experiences, importance of the IMR in choosing residency, and interests in learning IM and applying IM after graduation. Site characteristics included: extent of IM in the residency culture (e.g., faculty practicing IM consultations; IM consultation on site; an IM 4th year fellowship at the site); faculty characteristics (i.e., faculty leader IM fellowship trained; faculty leader with designated IM teaching time); and curriculum delivery (i.e., using the IMR plus other IM teaching/rotation/electives; monthly IM case conferences; resident IM clinical application).

## Results

Residents completing the curriculum and passing the final test were more likely to be female than male (88.9% vs. 60%; p=0.02) and US MDs (90.3%) or DOs (87.5%) than FMGs (52.9%) (p=0.023). There were no statistically significant differences on completion rates for the post-match survey items. Sites with the highest rates of resident course completion were those that had on-site IM consultations (p=<0.001), an IM 4th year fellowship (p=0.01), and a faculty leader with designated IMR time (p≤0.001). Medical knowledge test scores were significantly correlated with a greater number of faculty characteristics, culture characteristics, and total site characteristics but not with curriculum delivery.

## Conclusion

Characteristics of residents, residency faculty and culture are crucial to successful completion of a core curriculum in Integrative Medicine.

